# First report of *Staphylococcus pseudintermedius* ST71-SCC*mec* III and ST45-ΨSCC*mec*_57395_ from canine pyoderma in Argentina

**DOI:** 10.1186/s13104-023-06285-3

**Published:** 2023-02-23

**Authors:** Mariela E. Srednik, Claudia A. Perea, Gabriela I. Giacoboni, Jessica A. Hicks, Linda K. Schlater

**Affiliations:** 1 National Veterinary Services Laboratories, Animal and Plant Health Inspection Service, U.S. Department of Agriculture, Ames, IA U.S.A.; 2 Laboratorio de Bacteriología y Antimicrobianos, Departamento de Microbiología, Facultad de Ciencias Veterinarias, Universidad Nacional de La Plata, La Plata, Argentina

**Keywords:** *Staphylococcus pseudintermedius*, MRSP, SCC*mec*, MLST, ST71, ST45, Canine pyoderma

## Abstract

*Staphylococcus pseudintermedius* is an opportunistic pathogen commonly associated with skin infections in dogs. Twenty-three methicillin-resistant *S. pseudintermedius* (MRSP) isolated in Argentina from dogs with pyoderma were analyzed using whole genome sequencing (WGS) and classified into sequence types (ST) by multilocus sequence typing (MLST) and staphylococcal chromosome cassette *mec* (SCC*mec*) types.

Based on the WGS analysis, MLST, and SCC*mec* type results, we report for the first time in Argentina two MRSP strains, one each, belonging to ST71-SCC*mec* III and ST45-ΨSCC*mec*_57395_ from dogs with pyoderma. We also identified seven isolates with ST339, which had been previously reported in only two isolates in Argentina. Additionally, we identified ten MRSP isolates harboring variants of the SCC*mec* V found in *S. aureus*, seven SCC*mec* V (5C2&5) with two *ccr*C1 recombinases, and three SCC*mec* V (5C2) with one *ccr*C1 recombinase.

Our findings provide important insights into the evolution and geographic spread of these hypervirulent dominant clones that threaten the health of our companion animals and represent a significant risk for zoonotic infections.

## Introduction

*Staphylococcus pseudintermedius* is an important opportunistic pathogen in canine companions and is commonly associated with skin infections [1]. This bacterium is sporadically associated with human infections because it can be transmitted easily via close contact with animals, and it has the potential to cause severe disease [2]. Methicillin-resistant staphylococci of the intermedius group (SIG) emerged in canines in 1999 [3], and *S. pseudintermedius* was first described in 2005 [4]. Methicillin-resistant *S. pseudintermedius* (MRSP) has been spreading worldwide through the expansion and dissemination of dominant clonal lineages with specific genetic characteristics, including the sequence type (ST) 71 in Europe, ST68 in North America and ST45/ST112 in Asia [5, 6]. The first infection of MRSP in humans was reported in 2006 in Belgium [7] and the first MRSP isolated from a human patient in Argentina was reported in 2020 [8]. Furthermore, dominant clones are multi-drug resistant (MDR), suggesting that the spread of horizontally transferrable resistance genes is a contributing factor for the dissemination of certain sequence types [9].

Antimicrobial resistance patterns differ in the three most prevalent MRSP clonal lineages [5]. Clonal complexes (CCs) are groups of sequence types (STs) sharing at least six identical alleles of the seven *S. pseudintermedius* MLST genes (*ack*, *cpn*60, *fdh*, *pta*, *pur*A, *sar*, and *tuf*), with the primary founder being the ST with the largest number of single locus variants (SLVs) and all other strains diverge from the predicted clonal ancestor [10]. MRSP belonging to clonal complexes CC71 and CC68 often contain several genes that confer resistance to multiple antimicrobials in addition to the *mec*A gene located within the SCC*me*c cassette [11, 12]. For CC45, isolates often harbor resistance genes and mutations that make them resistant to almost all antimicrobials used in veterinary medicine [13].

In 2010, the global population structure of MRSP gradually started to change and it became more heterogenous than previously described, with evidence of dissemination through clonal expansion of MRSP dominant lineages over large distances [14]. In Europe, there was an apparent decrease of ST71 [6, 15, 16] with the emergence of two novel MRSP lineages (ST258 and ST496) of European and Australian origin [6, 17]. Likewise, ST71 clones began to spread worldwide over more distant locations and this clone has now been reported in Asia and in North and South America, with high prevalence in many countries in these regions. This change in the global population structure of *S. pseudintermedius* may be the consequence of importation from other countries due to the mobilization of animals and people across geographical locations [9, 18, 19]. In other parts of the world, the MRSP population appears to be more diverse. In Argentina, the MRSP population consists of genetically distinct STs not closely related to the more prevalent ST71 and ST68 lineages [20].

Staphylococcal chromosome cassette *mec* (SCC*mec*) typing is one of the molecular techniques currently used to understand the epidemiology and the clonal relationships of methicillin-resistant *S. aureus* (MRSA) strains [21]. Consequently, SCC*mec* typing for *S. pseudintermedius* has been progressively adapted from the work done for *S. aureus*. Existing reports of *S. pseudintermedius* SCC*mec* type III (previously described as II-III by Descloux et al. [22]) associated it with the European epidemic clone ST71, and ΨSCC*mec*_57395_ was significantly associated with ST45 [5, 11, 13]. To date, no knowledge exists regarding *S. pseudintermedius* belonging to the ST71 and ST45 clones in Argentina. Here we report for the first time in Argentina ST71-SCC*mec* III and ST45-ΨSCC*mec*_57395_.

## Main text

### Methods

#### Isolate selection

Thirty *S. pseudintermedius* isolates from dogs with pyoderma collected during 2016 from the Buenos Aires Metropolitan Area (Ciudad Autónoma de Buenos Aires, Gran Buenos Aires and La Plata, Argentina) were selected randomly from the strain collection of the Laboratory of Bacteriology and Antimicrobials, Department of Microbiology, Faculty of Veterinary Sciences, National University of La Plata, Argentina (Laboratorio de Bacteriología y Antimicrobianos, Departamento de Microbiología, Facultad de Ciencias Veterinarias, Universidad Nacional de La Plata, Argentina). Identification was confirmed by MALDI-TOF and whole genome sequencing (WGS) at the National Veterinary Services Laboratories (NVSL) in Ames, Iowa, U.S.A. Twenty-three *S. pseudintermedius* isolates were identified as methicillin-resistant (MRSP) due to the presence of the *mec*A gene, which encodes methicillin resistance, through WGS analysis (described below).

#### Whole genome sequencing and genomic analysis

Sequencing was performed with the Illumina MiSeq platform using 2 × 250 paired-end chemistry and the NexteraXT library preparation kit. Multilocus sequence typing (MLST) was determined using ABRicate (https://github.com/tseeman/abricate/) with the *S. pseudintermedius* PubMLST database, and new alleles and sequence types (STs) were submitted to PubMLST (http://pubmlst.org/spseudintermedius) for curation and number designation by Vincent Perreten (vincent.perreten@vetsuisse.unibe.ch). SCC*mec* types were determined using SCC*mec*Finder 1.2 [23] (https://cge.food.dtu.dk/services/SCCmecFinder-1.2/), a database with SCC*mec* types I through XII, including SCC*mec* IV and V subtypes (as of the preparation of this manuscript), based on those identified in *S. aureus*. For the predicted SCC*mec* types III and V, additional manual alignment/mapping was performed using the available reference sequences for these SCC*mec* types for *S. aureus* and *S. pseudintermedius* (AB03671.1, AM904732.1 for SCC*mec* type III; HE984157.2 for ΨSCC*mec*_57395_; and FJ544922.1, ERR175868, AB512767.1, AB505629.1, AB462393.1, AB121219.1 for SCC*mec* type V), using Geneious Prime v11.0.9 (Biomatters Ltd., NZ).

## Results

For the 23 MRSP isolates analyzed, a total of 14 sequence types (STs) were identified, five previously described: ST339 (n = 7), ST1412 (n = 3), ST71 (n = 2), ST45 (n = 1) and ST313 (n = 1); and nine newly identified STs (Table 1).

**Table 1:** Multilocus sequence types (MLST) and SCC*mec* types of methicillin resistant *Staphylococcus pseudintermedius* isolates obtained from dogs with pyoderma in Argentina

MLST	SCC*mec* type
ST339 (n = 7)	SCC*mec* V (5C2) (in only 2 isolates)
ST1412 (n = 3)	SCC*mec* V (5C2&5)
ST71 (n = 2)	SCC*mec* III (previously described as II-III)
ST45 (n = 1)	ΨSCC*mec*_57395_
ST313 (n = 1)	none
*Newly identified*	
ST2233 (n = 1), ST2234 (n = 1), ST2235 (n = 1), ST2242 (n = 1)	SCC*mec* V (5C2&5)
ST2261 (n = 1)	SCC*mec* V (5C2)
ST2243 (n = 1), ST2244 (n = 1), ST2236 (n = 1), ST2237 (n = 1)	none

SCC*mec*Finder successfully classified twelve isolates as SCC*mec* type IIIa (n = 2), SCC*mec* type V (5C2) (n = 3) and SCC*mec* type V (5C2&5) (n = 7). The remainder of the isolates could not be typed.

**Fig. 1:**
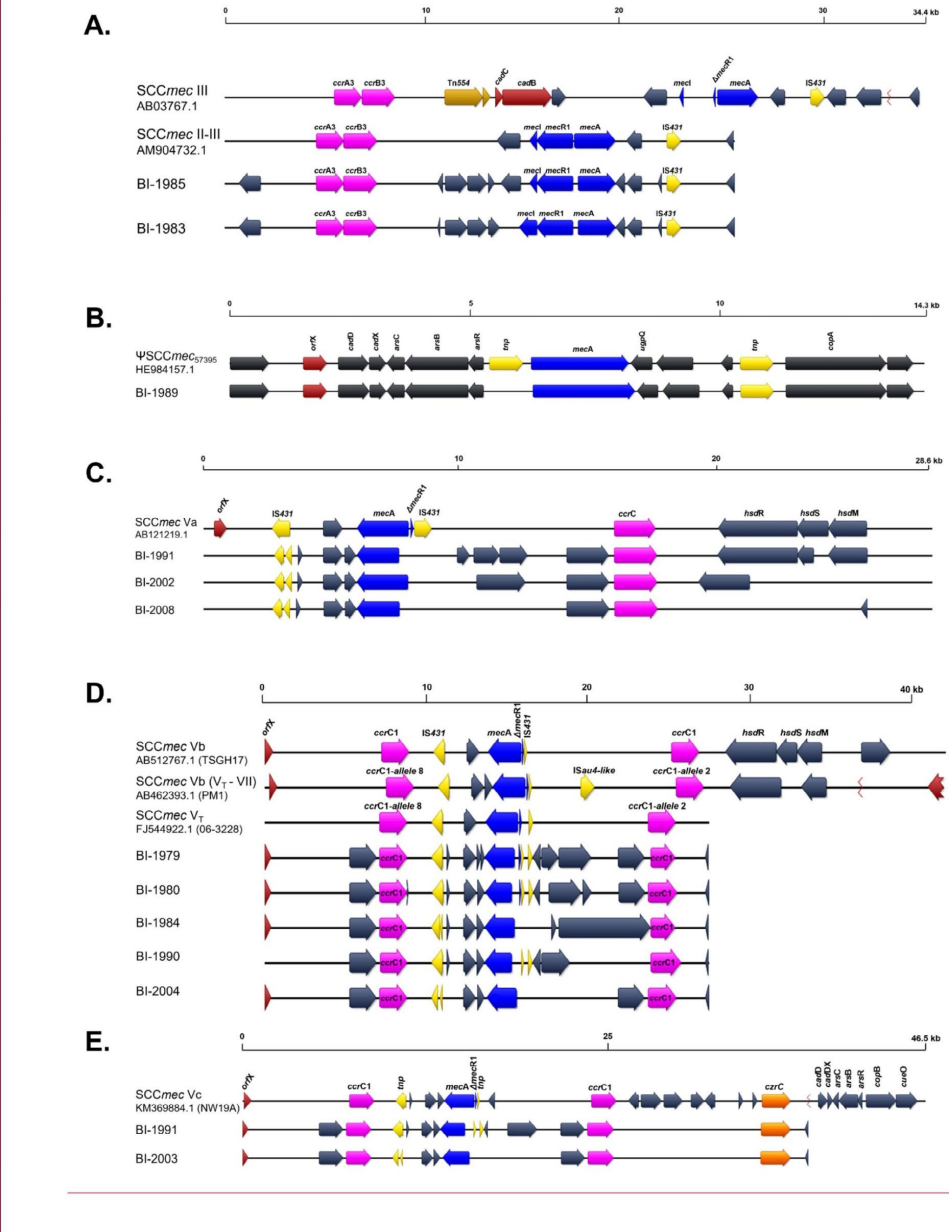
** A**, Alignment of *S. pseudintermedius* BI-1983 and BI-1985 SCC*mec* elements to *S. pseudintermedius* KM1381 (AM904732, first described as hybrid II-III) and *S. aureus* 85/2082 (AB037671.1, SCC*mec* III). **B**, Alignment of BI-1989 to *S. pseudintermedius* 57,395 (HE984157.2, ΨSCC*mec*_57395_). **C**, Alignment of SCC*mec* V (5C2) predicted elements for BI-1991, BI-2002 and BI-2008 to *S. aureus* SCC*mec* Va (5C2) [AB121219.1]. **D**, Alignment of SCC*mec* Vb (5C2&5) predicted elements for BI-1979, BI-1980, BI-1984, BI-1990, and BI-2004 to *S. aureus* Vb (5C2&5) [AB462393.1; AB512767.1] and *S. pseudintermedius* SCC*mec* V_T_ (FJ544922.1). E. Alignment of SCC*mec* Vc (5C2&5) predicted elements BI-1981 and BI-2003 to *S. aureus* Vc (5C2&5) [KM369884].

The two isolates classified as SCC*mec* type III belonged to ST71. These were mapped against the *S. pseudintermedius* KM1381 (AM904732.1) genome reference that harbors a hybrid SCC*mec* type II-III, described to be a combination of SCC*mec* II from *S. epidermidis* and SCC*mec* III from *S. aureus*, but lacking the cadmium resistance operon [22]. Both isolates showed high homology (99.9%) to this reference (Fig. 1A).

For one isolate identified as ST45,a SCC*mec* type could not be determined using SCC*mec*Finder, but alignment/mapping to HE984157.2 resulted in high homology (98.8%) classifying it as ΨSCC*mec*_57395_ (Fig. 1B).

Of the SCC*mec* type V, three were predicted as SCC*mec* type V (5C2), with only one *ccr*C1 recombinase, and seven were predicted as SCC*mec* type V (5C2&5), with two *ccr*C1 recombinases. When these 10 isolates were compared against SCC*mec* V subtype references (Va, Vb and Vc), isolates with SCC*mec* type V (5C2) (BI-1991, BI-2002, BI-2008) showed 79.4–90.6% homology to the *S. aureus* type Va (5C2) reference strain. The rest showed 84.8–99.8% homology to *S. pseudintermedius* 06-3228 (FJ544922.1) and *S. pseudintermedius* 23,929 (ERR175868), which are both references for *S. pseudintermedius* SCC*mec* V (5C2&5) [12, 24]. We classified five of these isolates as SCC*mec* Vb due to their homology with *S. aureus* AB462393.1 (Vb). Furhtermore, two of these SCC*mec* Vb (BI-1980, BI-1990) showed evidence of harboring a truncated *mec*R1 gene. Finally, we classified two isolates (BI-1991, BI-2003) as SCC*mec* Vc (5C2&5) because they harbored the *czr*C gene that is present in the SCC*mec* Vc but is absent in Vb. (Figure 1C, D and E).

## Discussion

This study is the first report of *S. pseudintermedius* ST71-SCC*mec* III and ST45-ΨSCCmec_57395_ in Argentina, obtained from a cohort of isolates recovered from dogs with pyoderma in the Buenos Aires Metropolitan Area in 2016. A previous study in Argentina described a population of MRSP from dogs with clinical disease that consisted of six genetically distinct STs: ST339, ST649, ST919, ST920, ST921, and ST922 [20]. Here, among 23 MRSP, ST339 (n = 7) was also identified, as well as an additional thirteen sequence types, including ST1412 (n = 3), ST71 (n = 2), ST45 (n = 1), ST313 (n = 1) and nine newly identified STs (ST2233-2237, ST2242-2244 and ST2261). These data contribute to the characterization of the population structure of MRSP in Argentina, which now includes two globally prevalent clones (ST71 and ST45). ST71 was initially described as the predominant clone in Europe but is now spread worldwide, whereas ST45 was described as the most prevalent clone in Asia [5]. Gagetti et al. [20] identified two isolates with sequence type ST339 in Argentina. The first MRSP recovered from a human patient in Argentina was ST1412 [8]. Interestingly ST1412 is a double locus variant of ST45, the sequence type that originated in Asia.

The ST71 clone has mainly been associated to SCC*mec* type III [11]. This SCC*mec*, first identified in 2005, was initially classified as a hybrid SCC*mec* II-III [22]. The distribution of this clone was primary found in Europe, but is now disseminated worldwide [23, 25]. The first report of an ST71 MRSP in South America was from a dog in Brazil in 2013 [26] and this study is the first report of this clone in Argentina. As in previous reports, the two isolates identified in this study as ST71 harbored SCC*mec* type III.

Pseudo (Ψ) SCC*mec* elements have been identified in *S. haemolyticus* with no evidence of *ccr* genes, but with a *mec* complex [27, 28]. A novel ΨSCC*mec*_57395_ was described in MRSP CC45 from companion animals in Thailand and Israel [13]. In Australia, MRSP belonging to ST45 was also associated to this novel ΨSCC*mec*_57395_ element [18]. Even though no particular SCC*mec* type is usually associated to MRSP-ST45 [25, 29], some reports identified ΨSCC*mec*_57395_ with this clone [13, 18]. The results from this study show evidence to also classify the MRSP-ST45 isolate from Argentina as an ST45-ΨSCC*mec*_57395_, making this the first report of this element in the country.

Lastly, almost half (10/23) of the isolates were predicted as SCC*mec* V. SCC*mec* V has been associated to different STs [5], and variation has been observed in SCC*mec* type V for *S. pseudintermedius* in comparison to *S. aureus.* Currently, this element is classified into three subtypes for *S. aureus*, according to Uehara [30]: Va (5C2), Vb (5C2&5) and Vc (5C2&5). To provide clarity, it’s important to mention how the classification for subtype Vb has evolved. Initially, it was classified as V_T_ (AB462393.1) [31]. Later, Black et al. [12] described a homologous SCC*mec* type V element in *S. pseudintermedius* (FJ44922.1), which only differed in a deleted section of a gene in *S. pseudintermedius* with respect to *S. aureus*. Then, Takano et al. [32] proposed reclassification of Vb to as SCC*mec* type VII. Finally, Perreten et al. [11], described an SCC*mec* V in *S. pseudintermedius* that was highly homologous to the previously named V_T_ or VII from *S. aureus*, which was designated as SCC*mec* V (5C2&5). In this study, three MRSP isolates, belonging to ST339, showed one *ccr*C1 recombinase only and were most homologous to SCC*mec* V (5C2). In contrast, the remaining seven MRSP isolates showed two *ccr*C1 recombinases and were most homologous to SCC*mec* V (5C2&5). Additionally, there was evidence to suggest that the *mec*R1 gene was truncated in two of these isolates (BI-1979 and BI-1980). Worthing et al. [18] reported similar results for the SCC*mec* V_T_ identified in their study. Prior to our study, SCC*mec* V (5C2&5) was the only SCC*mec* type reported in MRSP in Argentina [20].

## Conclusion

Using whole-genome sequencing we identified two MRSP isolates, one belonging to sequence type 71 and carrying staphylococcal cassette chromosome *mec* type III (ST71-SCC*mec* III), and the other belonging to sequence type 45 and carrying the ΨSCC*mec*_57395_ (ST45-ΨSCC*mec*_57395_), neither of which had been previously reported in Argentina. Even though these sequence types were first identified and distributed in Europe and Asia, respectively, our results support the current worldwide spread observed for these *S. pseudintermeius* clones. These findings highlight the importance of WGS for understanding the circulating populations of MRSP and the spread of multidrug-resistant *S. pseudintermedius* in companion animals, which can consequently have a significant impact on public health.

## Limitation

- Complete fragment coverage of the SCC*mec* elements was limited due to the inevitable gaps present in assemblies from short read technology, therefore fully closed genomes were not available.
- There are inconsistencies in the literature regarding nomenclature and classification of SCC*mec* elements, which makes interpretation and comparative analysis more complex.
- There is an evident need for a formal SCC*mec* nomenclature that includes SCC*mec* elements from *Staphylococcus pseudintermedius* and other *Staphylococcus* species.

## Data Availability

All sequence data was deposited in NCBI under BioProject PRJNA848756.

## Abbreviations

CC: Clonal complex
MDR: Multi-drug resistant
MLST: Multilocus sequence typing
MRSP: Methicillin-resistant Staphylococcus pseudintermedius
SCC*mec*: Staphylococcal cassette chromosome *mec*
SIG: Staphylococci of the intermedius group
ST: Sequence type
WGS: Whole genome sequencing
